# Effects of *Lactobacillus acidophilus* on the growth performance, immune response, and intestinal barrier function of broiler chickens challenged with *Escherichia coli* O157

**DOI:** 10.1016/j.psj.2021.101323

**Published:** 2021-06-10

**Authors:** Zhengke Wu, Kexin Yang, Anrong Zhang, Wenhuan Chang, Aijuan Zheng, Zhimin Chen, Huiyi Cai, Guohua Liu

**Affiliations:** ⁎ Feed Research Institute of Chinese Academy of Agricultural Science, Key Laboratory of Feed Biotechnology of Agricultural Ministry and Rural Affairs, Beijing 100081, China; † National Engineering Research Center of Biological Feed, Beijing 100081, China

**Keywords:** *Lactobacillus acidophilus*, broiler, intestinal barrier function, *Escherichia coli*

## Abstract

We studied the effects of *Lactobacillus acidophilus* (*L. acidophilus*) on the growth performance, intestinal morphology, barrier function, and immune response of broilers challenged with *Escherichia coli* O157 (***E. Coli***). A total of 360 1-day-old Cobb male broilers were tested in a 3 × 2 factorial arrangement with 3 dietary *L. acidophilus* levels (0, 5 × 10^8^ CFU/kg, and 10 × 10^8^ CFU/kg of diet) and 2 disease challenge treatments (control or *E. coli* challenged). Results showed that *E. coli* challenge decreased the ADG, ADFI, and BW of broilers from 15 to 21 d (*P* < 0.05), increased the jejunum intestinal wall thickness, and significantly increased the mortality rate. *E. coli* challenge significantly (*P* < 0.05) decreased the serum IgA and IgM contents and peripheral blood CD3+ T cell counts (*P* < 0.05), increased the serum CRP, DAO, and LPS levels at 21 d; upregulated the mRNA expression of iNOS, IL-8, IL-1β in the jejunum and iNOS in the spleen, and downregulated the occludin and ZO-1 mRNA expression in the ileum at 21 d compared with uninfected birds (*P* < 0.05). Dietary *L. acidophilus* supplementation consistently showed higher BW, ADG, ADFI, and jejunum and ileum V:C ratio at 14 d and 21 d in the presence and absence of *E. coli* challenge (*P* < 0.05). *L. acidophilus* supplementation reduced the mortality rate caused by *E. coli* challenge (*P* < 0.05), decreased the serum CRP, DAO, and LPS levels at 14 d and 21 d; upregulated the mRNA expression of occludin and ZO-1 in the jejunum and ileum, and downregulated the mRNA expression of iNOS, IL-8, and IL-1β in the jejunum in *E. coli* challenged birds at 21 d (*P* < 0.05). Dietary supplementation with *L. acidophilus* can improve the growth performance, intestinal health, and survival of broilers challenged with *E. coli*.

## INTRODUCTION

*Escherichia coli* (***E. coli***) infection is common in commercial animal breeding situations on a world basic (Reda et al., 2019). In poultry, *E. coli* infection causes severe health issues and reduces production (Landman et al., 2015; Ronco et al., 2017). Infected chickens and contaminated poultry meat or eggs are important carriers of *E. coli*, which is a threat to human health. *E. Co, 51li* O157 is a common serotypes of *E. coli* bacteria reported worldwide. It is important to control *E. coli* outbreaks in the poultry industry. For many decades, antibiotics have typically been used to prevent infections of *E. coli* and other pathogens in animals and humans (Kabir and Lutful, 2009). However, excessive use of antibiotics in animal feed has increased the number of drug-resistant pathogen strains. This has led to animals harboring these strains being reservoir hosts of antibiotic resistance genes (Gao et al., 2017). The intestinal microbiota of domestic animals might then be a reservoir for resistance genes in pathogens that may be transmitted to humans (Smet et al., 2011). Many countries have banned the use of antibiotics in animals feed as growth promoters (Cogliani et al., 2011). The European Union-wide ban on the use of antibiotic growth promoters in farm animals in 2006 was an initial step in dealing with the antibiotic resistance issue. In China, the use of antibiotics as growth promoters has been banned since July 1, 2020. Enteric diseases have seen become a prime concern in the poultry industry after the exclusion of antibiotics. To maintain a healthy intestinal status and improve animal growth without using antibiotics, there are growing demands for safe, effective, and economical antibiotic alternatives.

Probiotics are nonpathogenic, microorganisms that have a beneficial effect on the growth performance, immune function, intestinal microbiology or physiology of the host when administered in adequate amounts (Gill, 2008; Hou et al., 2016). Probiotics can be provided as a live microbial feed supplement, also known as direct fed microbials, in the poultry diet or administered to the developing embryo using in vivo feeding technology (Pender et al., 2016). The addition of probiotics to poultry feed has been studied to improve nutrient digestibility and absorption, modulate the intestinal microbiota balance, keep the intestinal tract healthy, improve the immune response, and relieve stress (Patterson and Burkholder, 2003; Revolledo et al., 2006; Prado-Rebolledo et al., 2016; Li et al., 2018). These studies demonstrated that probiotics may provide a potential alternative strategy to the use of antibiotics in poultry feed. *Lactobacillus acidophilus* (***L. acidophilus***) is a species of gram positive bacteria in the genus *Lactobacillus* and is widely used as a probiotic for humans and animals (Li et al., 2018). *L. acidophilus* can inhibit pathogen invasion (Lin et al., 2008) and modulate immunity response in vitro and and in vivo (Lin et al., 2010). *L. acidophilus* can produce bacteriostatic bacteriocin-like compounds as well as acids which decrease the gut pH (Chateau et al., 2010). Adding *L. acidophilus* to poultry feed helps prevent the proliferation of pathogenic bacteria and regulates the intestinal flora through competitive exclusion and antagonism (Fuller, 1989; Frece et al., 2009). These studies demonstrated that *L. acidophilus* has the potential to control animal pathogens. However, it is unclear whether dietary *L. acidophilus* supplementation can improve the intestinal health and growth performance of broilers in an *E. coli* challenge model. Therefore, our objective was to evaluate the efficacy of *L. acidophilus* in controlling *E. coli* infection in broiler chickens by determining its effect on growth performance, intestinal morphology, immune responses, and cytokine mRNA expression.

## MATERIALS AND METHODS

### Animal Ethics Statement

All animal management and experimental procedures for this study were approved by the Animal Ethic Committee of the Chinese Academy of Agricultural Sciences and performed according to the guidelines for animal experiments set by the National Institute of Animal Health.

### Experimental Design and Animal Management

A 3 × 2 factorial arrangement of treatments was used in a completely randomized design to investigate the effects of 3 levels of *L. acidophilus* supplementation (0, 5 × 10^8^ CFU/kg, and 10 × 10^8^ CFU/kg of diet) and 2 disease treatments (control or *E. coli* challenged). A total of 360 one-day-old Cobb male broilers were allocated to 6 treatments in a completely randomized design ensuring the average body weight was the same for all the treatments. The 6 treatment groups were as follows: group 1, control diet, uninfected; groups 2 and 3, *L. acidophilus* supplemented diet (5 × 10^8^ CFU/kg and 10 × 10^8^ CFU/kg of diet), uninfected; group 4, control diet, infected with *E. coli*; groups 5 and 6, *L. acidophilus* supplemented diet (5 × 10^8^ CFU/kg and 10 × 10^8^ CFU/kg of diet), infected with *E. coli.* Each group had 6 replicate cages with 10 birds per cage. All birds were reared under identical conditions according to the Cobb Broiler management guide. Birds were raised on floor monolayer stainless steel cage (1.2 × 0.9 × 0.7 m) and allowed free access to clean water and experiment feed during the entire experiment period. During the first 3 d, the ambient temperature in the room was maintained at 35°C and was gradually reduced reaching 25°C at 21 d of age. The photoperiod was 17:7 h (L:D).

### Diets and *L. Acidophilus* Supplementation

Chickens were fed an antibiotic-free corn-soybean meal basal diet in the form of mash. All nutrients were formulated to meet or exceed the feeding standard of China (NY/T 2004) for broilers. *L. acidophilus* (CGMCC 14437) used in this study was provided by the China General Microbiological Culture Collection Center (Beijing, China) and it was added to the basal diet in the form a bacterial suspension, providing 5 × 10^8^ CFU/kg and 10 × 10^8^ CFU/kg of diet. To ensure the homogeneity of the additives, approximately 20 kg of the basal diet was mixed with the calculated amount of bacteria using a 60-L horizontal mixer. Fresh experimental diets were produced manually every 3 d.

*L. acidophilus* was stored at –80°C in their media containing 10% glycerol (Solarbio, China). Before the experiment, *L. acidophilus* was propagated in Mane Rogosae Sharpe (MRS, Aobox, China) agar at 37°C for 18 h. MRS broth was aseptically inoculated at 37°C for 21 h and centrifuged at 4°C for 10 min at 2,000 *g*. After removal of the supernatant, the bacteria precipitate was washed twice with phosphate-buffered saline (**PBS**) and was suspended in PBS medium at a final concentration of 1 × 10^9^ CFU/mL.

### E. *Coli* Challenge

The *E. coli* O157(CAU0771) was obtained from the China Veterinary Culture Collection Center, the China Institute of Veterinary Drug Control (Beijing, China). Briefly, *E. coli* O157 was cultured under aerobic condition on Luria-Bertani agar for 12 h at 37℃, and then aseptically inoculated into cooked Luria-Bertani meat medium and incubated aerophile for 18 h at 37℃, 200 r/min. Birds in the challenge groups were orally perfused once daily with actively growing *E. coli* O157 (3 × 10^8^ cfu/mL, 0.5 mL per bird) during d 9 to 11, while the unchallenged birds were perfused with the same volume of sterilized cooked Luria-Bertani meat medium (Aoboxing, China).

### Measurement of Growth Performance

Birds’ body weight and feed intake were group measured by cage at 1, 14, and 21 d of age. Average daily gain (**ADG**), average daily feed intake (**ADFI**), and feed conversion ratio (feed: body weight gain, **FCR**) were calculated during the different period (1–14 d and 15–21 d).

Mortality was recorded (along with the birds’ body weight and cause of death, if known) during d 9 to 21. Birds were checked at least once daily and birds were euthanized by intravenous injection of pentobarbitone if they were in an unusual state (e.g., lame, visibly smaller than cage-mates, ruffled feathers, and lethargic), or had a serious injury or disease (e.g., broken wing and severe diarrhea). Mortality is reported as the percentage of the birds culled and found dead.

### Peripheral Blood Mononuclear Cell Isolation and Analysis by Flow Cytometry

At 21 d, peripheral blood mononuclear cell (**PBMC**) was isolated from peripheral blood samples using Ficoll density centrifugation according to the method of Tan et al. (2015)*.* Briefly, wing venous blood was collected and layered on top of the separation medium (Histopaque 1077; Sigma Chemical Company, Boston, MA) in tubes (1:1) and centrifuged at 200 *g* for 30 min (25°C). Then the PBMC at the plasma–Ficoll interface were collected. Cold RPMI-1640 medium (containing 5.0% inactivated fetal bovine serum, 100 U (0.0599 mg) penicillin/mL, 100 mg streptomycin/mL, and 10 mM-HEPES) was added to the tube containing the PBMC. After that, the cells were washed 3 times with cold RPMI-1640 medium by centrifugation at 100 *g* for 10 min (4°C). Then the samples were measured and analyzed using a FACSort flow cytometer (Becton Dickinson, Mountain View, CA) and Cell Quest software. First, the PBMC were incubated with specific antileucocyte monoclonal antibodies. The monoclonal antibodies (CD3-PE, CD4-APC, CD8-FITC) were purchased from Southern Biotech (Birmingham, AL). The cells were incubated with antibody at 4°C for 30 min then washed 3 times with PBS. Then samples were analyzed using the flow cytometer. Lymphocyte and monocyte subpopulations were gated based on forward and side-scatter characteristics, and the PBMC subpopulation counts were calculated based on 100 gated lymphocytes.

### Measurement of Serum Immune Globulin, C-Reactive Protein, Diamine Oxidase, and Lipopolysaccharide Content

Blood samples from 6 birds per group (1 subsample /replicate cage) were collected into a disposable vacuum blood tubes containing procoagulant and separation gel at the end of the experiment period (21 d). All blood samples were centrifuged at 3,000 rpm for 10 min and the serum part was transferred into three separate tubes and stored at −20°C for analysis. The blood serum samples of immune globulin A (**IgA**, kit No. E027-1-1), immune globulin M (**IgM**, kit No. E025-1-1), C-reactive protein (**CRP**, kit No. E024-1-1), diamine oxidase (**DAO**, kit No. A088-1-1), and endotoxin lipopolysaccharide (**LPS**, kit No. E039-1-1) were determined according to the protocols of the kits of Nanjing Jiancheng Bioengineering Institute (Nanjing, China).

### Total RNA Extraction and Quantitative Real-Time PCR for Measuring Related Gene Transcript Levels in the Jejunum, Ileum, and Spleen

At 3 and 10 d postinfection (at 14 and 21 d of age), 1 bird from each cage was euthanized by intravenous injection of pentobarbitone. The middle segments (2 cm in length) of jejunum and ileum of each bird were excised and snap-frozen in liquid nitrogen and stored at −80°C until further mRNA analysis. At 10 d postinfection (at 21 d of age), spleen samples of each bird were also excised and snap-frozen in liquid nitrogen and stored at −80℃ until further mRNA analysis .

Total RNA was isolated from snap-frozen jejunum, ileum and spleen tissue samples (50 mg) by using TRIzol reagent (Invitrogen Life Technologies, Carlsbad, CA) according to manufacturer instructions. The concentration and purity of the total RNA were measured in a Microplate Readers (Multiskan Sky, 1.00.55, Thermo Fisher Scientific, Waltham, MA) using the 260:280 nm absorbance ratio. The absorption ratio (OD260/ OD280) of total RNA samples ranged between 1.8 and 2.0 and was considered to be of qualified purity. First, complementary DNA (**cDNA**) was synthesized from 1 μg of total RNA using Fast King RT Kit (Tiangen, China, kit No. KR116) according to the manufacturer's instructions and stored at −20°C until further processing. Quantitative real-time PCR was performed by Applied Biosystems Bio-Rad Real-Time PCR system (Bio-Rad, Carlsbad, CA) and a Premix Ex Taq with SYBR Green (Tiangen, China, kit No. FP205). Reactions were conducted in a 20-μL reaction mixture which contain 10.0 μL of 2 × SYBR PreMix plus, 1.0 μL of cDNA, 0.6 μL of each primer (10 mmol/L), and 7.8 μL of RNase-free water. For PCR, the thermocycle protocol lasted for 15 min at 95°C, followed by 40 cycles of 10 s denaturation at 95°C, 34 s annealing/extension at 60°C, and then a final melting curve analysis to monitor purity of the PCR product. Oligonucleotide primers for chicken iNOS, IL-6, IL-8, IL-1*β*, NF-kB, occludin, ZO-1, claduin, and chicken β-actin (Table 1) were designed based upon sequences available from public databases using Primer Express 5.0. The average gene expression of each sample relative to that of β-actin was calculated using the 2^−ΔΔCt^ method as previously described (Livak and Schmittgen, 2001).

**Table 1:** Primers used for relative real-time polymerase chain reaction.

Gene name	Forward primer sequence (5′ to 3′)	Reverse primer sequence (5′ to 3′)	GenBank number
β*-*action	GAGAAATTGTGCGTGACATCA	CCTGAACCTCTCATTGCCA	NM_205518.1
iNOS	CAGCTGATTGGGTGTGGAT	TTTCTTTGGCCTACGGGTC	NM_204961.1
IL-6	AGGGCCGTTCGCTATTTGAA	CAGAGGATTGTGCCCGAACT	XM_015281283.2
IL-8	ATGAACGGCAAGCTTGGAGCTG	TCCAAGCACACCTCTCTTCCATCC	NM_205498.1
IL-1β	ACTGGGCATCAAGGGCTA	GGTA GAAGATGAAGCGGGTC	XM_015297469
NF-kB	GTGTGAAGAAACGGGAACTG	GGCACGGTTGTCATAGATGG	XM_025145278.1
Occludin	ACGGCAGCACCTACCTCAA	GGGCGAAGAAGCAGATGAG	XM_025144248
ZO-1	CTTCAGGTGTTTCTCTTCCTC	CTGTGGTTTCATGGCTGGATC	XM_015278981
Claudin-1	CATACTCCTGGGTCTGGTTGGT	GACAGCCATCCGCATCTTCT	NM_001013611.2

Abbreviations: iNOS, inducible nitric oxide synthase; IL, interleukin; NF-kB, nuclear factor kappa; ZO-1, zona occludens protein-1.

### Jejunum and Ileum Morphology

At 14 and 21 d, 1 bird from per cage was euthanized by intravenous injection of pentobarbitone. The intestinal samples were processed following a previously published protocol (Gungor and Erener, 2020). Intestinal segment samples (approximately 2–3 cm in length) of jejunum and ileum were excised and flushed with 0.9% saline to remove the feed contents and fixed in 10% neutral-buffered formalin for histology. Duodenal tissues were embedded in paraffin, and 20 cross-sections 2-mm thick of each sample were cut. Each of 5 semiserial cuts were placed on one microscopic slide and stained with Alcian blue and Periodic Acid-Schiff reagent (Solarbio G1281, Beijing, China). Ten well-oriented villi of each sample were selected to measure duodenal morphology. Villus height was measured from the tip to the villus-crypt junction of each villus. Afterward, the thickness of the jejunal and ileal wall (including the mucosa, submucosa, muscularis, and serosa) was measured. Crypt depth was calculated based on the distance from the junction to the basement membrane of the epithelial cells at the bottom of each 227crypt. Then, the ratio of villus height and crypt depth (**V/C**) was calculated.

### Statistical Analysis

All data in this experiment were analyzed with SPSS version 17.0 (SPSS Inc., Chicago, IL). Data in tables represent the mean and standard error (**SE**) of 6 replicates. The main effects of *L. acidophilus* supplementation, *E. coli* challenge, and their interactive effects were analyzed as a 3 × 2 factorial arrangement of treatments. Duncan's multiple comparison test was used to separate means when interactive effects significantly differed. Results were also analyzed by one-way ANOVA, and were subjected to ground tables of the GraphPad Prism 7 (GraphPad Software, San Diego, Inc., CA). When a significant difference was observed, Duncan's multiple comparison was used to compare the differences among the 6 groups. Statistical significance was considered to be a *P* < 0.05, and a probability of *P* < 0.05 < *P* < 0.10 was considered as indicating a trend.

## RESULTS

### Growth Performance and Mortality Parameters

**Figure 1:**
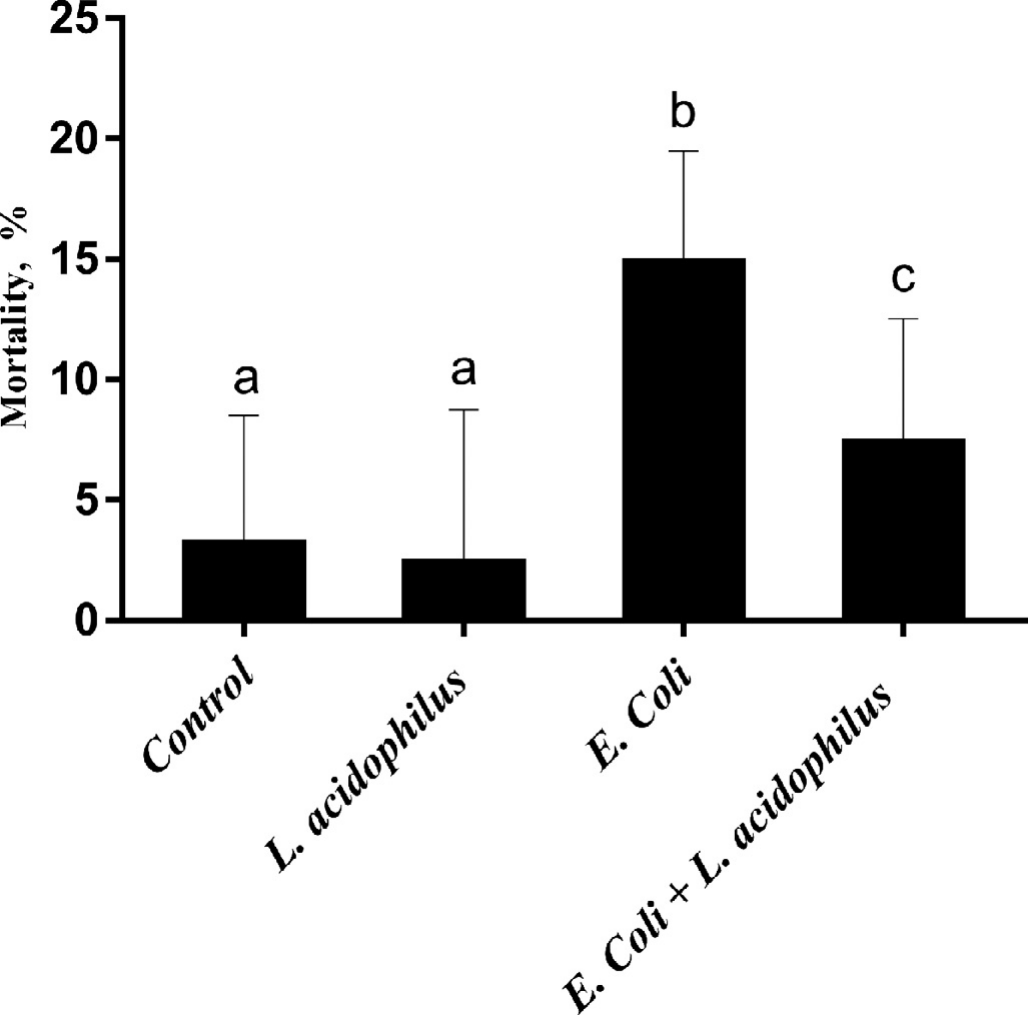
Effect of *E. Coli* challenge and *L. acidophilus* on mortality rates in broiler chickens during d 9–21.

The growth performance and mortality rates of broiler chickens are present in Table 2 and Figure 1. Dietary *L. acidophilus* supplementation was associated with higher BW, ADG, ADFI, and FCR of broilers at different time periods (*P* < 0.05). Compared with uninfected groups, *E. coli* challenge did not significantly affect BW, ADG, ADFI, and FCR of broilers during 1 to 14 d (*P* >0.05), but decreased the ADG (*P* < 0.05), ADFI (*P* < 0.05), and BW (*P* = 0.083) of broilers from 15 to 21 d. No significant interaction (*P* > 0.05) in growth performance between *E. coli* challenge and *L. acidophilus* supplementation was observed. The *E. coli* challenge significantly increased the mortality rate (*P* < 0.05) of broilers at 21 d. *L. acidophilus* supplementation significantly reduced the mortality rate of *E. coli* challenged broilers (*P* < 0.05).

**Table 2:** Effect of *L. acidophilus* on growth performance of broilers challenged with *E. coli.*

		d 1–14				d 15–21			
*L. acidophilus*	*E. coli* challenge^2^	BW, g	ADG, g/d	ADFI, g/d	FCR	BW, g	ADG, g/d	ADFI, g/d	FCR
0	−	377.37^a^	25.61^a^	34.68^ab^	1.36^a^	762.71^ab^	54.20^ab^	80.29^ab^	1.48
5 × 10^8^ CFU/kg	−	412.50^b^	28.32^b^	36.77^a^	1.30^b^	829.63^c^	59.72^c^	89.75^c^	1.51
10 × 10^8^ CFU/kg	−	415.45^b^	28.57^b^	36.75^a^	1.29^b^	851.14^c^	60.70^c^	89.78^c^	1.48
0	+	369.56^a^	25.12^a^	33.65^b^	1.34^ab^	751.33^a^	50.68^a^	74.59^a^	1.47
5 × 10^8^ CFU/kg	+	403.92^b^	27.66^b^	36.07^a^	1.31^ab^	800.81^ab^	55.32^b^	83.21^bc^	1.51
10 × 10^8^ CFU/kg	+	404.72^b^	27.68^b^	36.47^a^	1.32^ab^	813.73^bc^	57.89^bc^	84.57^bc^	1.46
SEM		6.39	0.32	0.34	0.01	8.89	0.78	1.21	0.01
Main effects									
*E. coli challenge*									
Positive		401.77	27.50	36.07	1.314	814.50	58.21^a^	86.61^a^	1.489
Negative		392.73	26.64	35.36	1.330	788.62	54.63^b^	80.79^b^	1.480
*L. acidophilus* supplementation									
0		373.46^a^	25.10^a^	34.11^a^	1.362^a^	757.01^a^	52.44^a^	77.44^a^	1.477
5 × 10^8^ CFU/kg		408.21^b^	27.99^b^	36.42^b^	1.302^b^	815.22^b^	57.52^b^	86.45^b^	1.507
10 × 10^8^ CFU/kg		410.08^b^	28.12^b^	36.61^b^	1.303^b^	832.43^b^	59.29^b^	87.18^b^	1.470
*P*-value^1^									
* E. coli challenge*		0.174	0.112	0.243	0.346	0.083	0.005	0.003	0.760
* L. acidophilus*		0.001	0.001	0.003	0.007	0.001	0.001	0.001	0.450
*E. coli challenge* × *L. acidophilus*		0.982	0.961	0.832	0.814	0.760	0.853	0.953	0.951

^a,b,c^Means within the same column with different superscripts differ significantly (*P* < 0.05).

Abbreviations: ADG, average daily gain; ADFI, average daily feed intake; BW, body weight; FCR, feed conversion ratio, g of feed intake / g of BW gain, g/g.

1 *P*-values represent the main effect of the *L. acidophilus*, the main effect of *E. coli* challenge, and the interaction between the dietary *L. acidophilus* and *E. coli* challenge.

2 Without *E. coli* challenge; +, with *E. coli* challenge.

### Intestinal Morphology

There was an interaction effect (*P* < 0.05) on the jejunum V:C ratio between *L. acidophilus* supplementation and *E. coli* challenge (Table 3). Broilers fed diets supplemented with *L. acidophilus* (5 × 10^8^ CFU/kg) had a significant increase of the jejunum V/C ratio and ileum villus height at 14 d but this favorable change disappeared when the broilers were infected with *E. coli*. Based on the main effects analysis, a decreasing trend in ileum villus height of *E. coli* challenged birds was observed at 14 d (*P* = 0.100).

**Table 3:** Effect of *L. acidophilus* on jejunum and ileum histomorphology of broilers infected with *E. coli* on d 14.

		Jejunum				Ileum			
*L. acidophilus*	*E. coli* challenge^2^	Villus height, μm	Crypt depth, μm	V/C	Intestinal wall thickness, μm	Villus height, μm	Crypt depth, μm	V/C	Intestinal wall thickness, μm
0	−	1094.52	201.28	5.33^a^	200.93	810.90^b^	205.89	4.06	217.56
5 × 10^8^ CFU/kg	−	1070.26	159.94	6.71^b^	188.77	965.77^a^	211.50	4.64	191.63
10 × 10^8^ CFU/kg	−	974.55	211.44	4.65^a^	199.44	810.77^b^	223.84	3.70	221.98
0	+	994.73	200.42	5.24^a^	189.40	794.94^b^	193.17	4.22	227.39
5 × 10^8^ CFU/kg	+	874.33	179.03	5.04^a^	188.78	820.96^b^	219.49	3.79	218.98
10 × 10^8^ CFU/kg	+	974.09	171.21	6.03^ab^	181.27	790.52^b^	204.96	4.32	221.85
SEM		33.21	7.99	0.23	6.04	20.57	8.18	0.18	5.83
Main effects									
*E. coli* challenge									
Negative		1046.44	190.88	5.57	196.38	862.48	216.24	4.21	209.78
Positive		947.71	183.55	5.44	186.48	802.14	205.87	4.11	222.74
*L. acidophilus* supplementation									
0		1044.62	200.85	5.29	195.17	802.92^a^	199.83	4.14	222.48
5 × 10^8^ CFU/kg		972.29	169.48	5.88	188.78	893.36^b^	209.25	4.32	204.39
10×10^8^ CFU/kg		974.31	191.33	5.34	190.35	800.64^a^	204.01	4.10	221.90
*P*-value^1^									
* E. coli* challenge		0.151	0.651	0.578	0.450	0.100	0.564	0.811	0.294
* L. acidophilus*		0.599	0.277	0.613	0.913	0.098	0.642	0.832	0.407
*E. coli* challenge × *L. acidophilus*		0.510	0.322	0.035	0.849	0.323	0.904	0.275	0.615

^a,b^Means within the same column with different superscripts differ significantly (*P* < 0.05).

1 *P*-values represent the main effect of the *L. acidophilus*, the main effect of *E. coli* challenge, and the interaction between the dietary *L.acidophilus* and *E. coli* challenge.

2 Without *E. coli* challenge; +, with *E. coli* challenge.

Compared with birds fed basal diet, *L. acidophilus* treatment tended to increase the ileum V:C ratio at 21 d (Table 4, *P* = 0.052) and an interaction effect was also observed between *L. acidophilus* supplementation and *E. coli* challenge (*P* < 0.05). *E. Coli* challenge significantly increased the jejunum intestinal wall thickness at 21 d compared with unchallenged birds and *E. coli* challenge birds fed with *L. acidophilus* (10 × 10^8^ CFU/kg).

**Table 4:** Effect of *L. acidophilus* on jejunum and ileum histomorphology of broilers infected with *E. coli* on d 21.

		Jejunum				Ileum			
*L. acidophilus*	*E. coli* challenge^2^	Villusheight, μm	Crypt depth, μm	V/C	Intestinal wall thickness, μm	Villusheight, μm	Crypt depth, μm	V/C	Intestinal wall thickness, μm
0	−	979.70^b^	214.86	4.73^a^	175.77^b^	634.43	187.04	3.47^b^	227.64
5 × 10^8^ CFU/kg	−	962.46^b^	203.20	4.96^a^	177.02^b^	538.19	148.81	3.71^b^	191.48
10 × 10^8^ CFU/kg	−	1247.91^a^	236.21	5.55^b^	188.01^ab^	706.71	156.32	4.56^a^	214.51
0	+	1182.63^ab^	237.89	5.08^ab^	195.47^a^	640.99	181.92	3.58^b^	239.69
5 × 10^8^ CFU/kg	+	1105.64^ab^	213.19	5.31^b^	199.58^a^	703.35	175.70	3.99^ab^	233.33
10×10^8^ CFU/kg	+	1136.93^ab^	187.96	6.33^c^	187.11^ab^	649.71	180.06	3.62^b^	209.20
SEM		36.74	9.10	0.23	5.53	24.00	6.74	0.10	8.11
Main effects									
*E. coli challenge*									
Negative		1063.36	218.09	5.07	180.26	626.44	164.06	3.91	211.21
Positive		1141.73	213.02	5.57	194.06	664.68	179.23	3.73	227.41
*L. acidophilus* supplementation									
0		1081.16	226.38	4.905^a^	185.62	637.71	184.48	3.521^a^	233.67
5 × 10^8^ CFU/kg		1034.05	208.19	5.135^a^	188.30	620.77	162.25	3.848^ab^	212.40
10 × 10^8^ CFU/kg		1192.42	212.09	5.938^b^	187.56	678.21	168.19	4.088^b^	211.85
*P*-value^1^									
*E. coli challenge*		0.267	0.791	0.305	0.086	0.432	0.279	0.325	0.340
*L. acidophilus*		0.195	0.700	0.021	0.172	0.616	0.395	0.052	0.480
*E. coli challenge* × *L. acidophilus*		0.176	0.295	0.916	0.127	0.170	0.577	0.023	0.515

^a,b,c^Means within the same column with different superscripts differ significantly (*P* < 0.05).

1 *P*-values represent the main effect of the *L.acidophilus*, the main effect of *E. coli* challenge, and the interaction between the dietary *L. acidophilus* and *E. coli* challenge.

2 Without *E. coli* challenge; +, with *E. coli* challenge.

### Immune Cell Phenotypes

**Figure 2:**
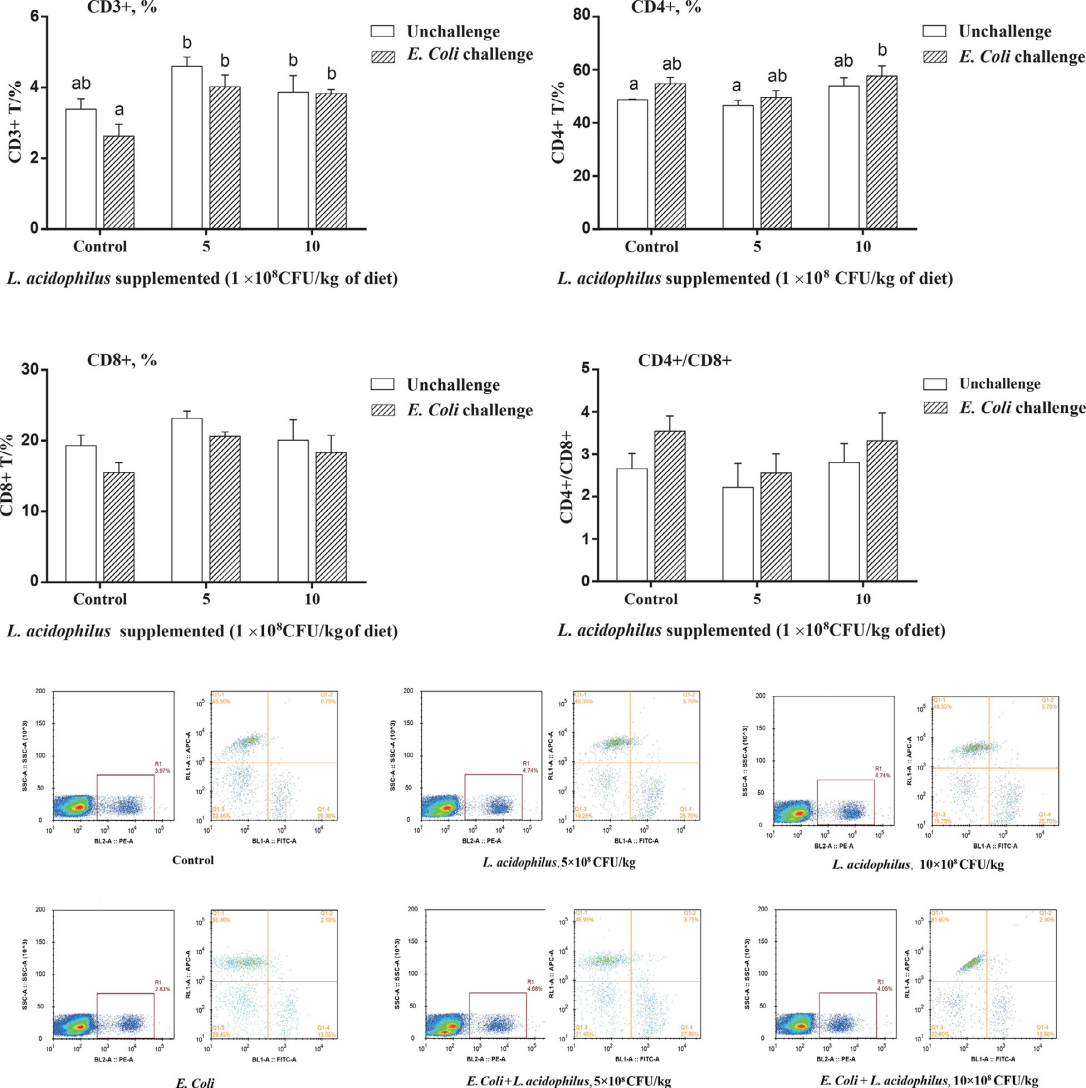
The distribution of CD3+, CD4+, and CD8+ of lymphocytes isolated from peripheral blood in 21-day-old chickens treated with control diet , 5 × 10^8^ CFU/kg L. acidophilus, 10 × 10^8^ CFU/kg L. acidophilus, *E. Coli, E. Coli* + 5 × 10^8^ CFU/kg L. acidophilus, *E. Coli* + 10 × 10^8^ CFU/kg L. acidophilus, The collected cells from each treatment were stained with mice anti-chicken monoclonal antibodies (PE-labeled anti-CD3, PE-labeled anti-CD4, and PE-labeled anti-CD8) and analyzed in a flow cytometer. The graphs show the ratio of CD3+/CD4+ (E) and CD3+/CD8+ (F) divided by the total T cells of peripheral blood mononuclear cells. Each bar represents mean ± standard error (SE) of 6 replicates. Means with no common letter differ significantly (*P* < 0.05).

The immune cell subpopulation data on d 21 are shown in Figure 2. *E. coli* challenge significantly decreased (*P* < 0.05) the CD3+ T cell counts compared with unchallenged birds and challenged birds fed with *L. acidophilus*. The subpopulation of CD4+ T cells subpopulation were highest in *E. coli* challenge birds fed 10 × 10^8^ CFU/kg of *L. acidophilus.* No significant differences (*P* > 0.05) were observed for CD8+T cells subpopulation's changes between groups. In addition, the ratio of CD4+ T cells and CD8+ T cells in the peripheral blood lymphocyte subpopulations were not significantly different.

### Serum IgA, IgM, CRP, DAO, and LPS Content

The serum index was measured to estimate the humoral immunity (Table 5) and intestinal barrier function (Table 6). No significant interaction effect was observed between *L. acidophilus* supplementation and *E. coli* challenge in terms of serum specific IgA and IgM content of broilers. Compared with the unchallenged birds, *E. coli* challenge significantly (*P* < 0.05) resulted in an increase in the serum concentration of IgA and IgM at the early stage of infection at d 14 (at 3 d postinfection), but the serum IgA and IgM level were decreased and significantly lower than unchallenged birds at 21 d (at 10 d postinfection). Large increases in serum IgA and IgM were observed in *E. coli* challenged birds fed with *L. acidophilus* (10 × 10^8^ CFU/kg) compared with infected birds at 21d.

**Table 5:** Effect of *L. acidophilus* on serum immune globulin content of broilers infected with *E. coli* on d 14 and d 21.

		d 14		d 21	
*L. acidophilus*	*E. coli* challenge^1^	IgA^2^, g/L	IgM^3^, g/L	IgA, g/L	IgM, g/L
0	−	1.23^a^	1.96^abc^	3.22^b^	3.21^b^
5 × 10^8^ CFU/kg	−	1.48^a^	1.81^ab^	3.26^b^	3.62^b^
10 × 10^8^ CFU/kg	−	1.63^ab^	1.72^a^	2.90^b^	3.32^b^
0	+	1.98^ab^	2.29^c^	2.19^a^	2.18^a^
5 × 10^8^ CFU/kg	+	2.46^b^	2.10^bc^	3.47^b^	3.04^b^
10 × 10^8^ CFU/kg	+	2.31^b^	2.05^abc^	2.46^ab^	2.69^ab^
SEM		0.16	0.06	0.17	0.15
Main effects					
*E. coli* challenge					
Negative		1.49^a^	1.830^a^	3.458^a^	3.380^a^
Positive		2.25^b^	2.147^b^	3.127^b^	2.637^b^
*L. acidophilus* supplementation					
0		1.60	2.126	2.707^a^	2.69
5 × 10^8^ CFU/kg		1.97	1.954	3.362^b^	3.33
10 × 10^8^ CFU/kg		1.97	1.885	2.678^a^	3.00
*P*-value^4^					
*E. coli* challenge		0.010	0.003	0.043	0.009
*L. acidophilus*		0.467	0.113	0.069	0.156
*E. coli* challenge × * L. acidophilus*		0.898	0.982	0.615	0.733

^a,b,c^Means within the same column with different superscripts differ significantly (*P* < 0.05).

1 Without *E. coli* challenge; +, with *E. coli* challenge.

2 IgA, immune globulin A.

3 IgM, immune globulin M.

4 *P*-values represent the main effect of the *L. acidophilus*, the main effect of *E. coli* challenge, and the interaction between the dietary *L. acidophilus* and *E. coli* challenge.

**Table 6:** Effect of *L. acidophilus* on serum endotoxin content serum of broilers infected with *E. coli* on d 14 and d 21.

		d 14			d 21		
*L. acidophilus*	*E. coli* challenge^1^	CRP^2^ (mg/L)	DAO^3^ (U/L)	LPS^4^ (EU/mL)	CRP (mg/L)	DAO (U/L)	LPS (EU/mL)
0	−	3.29^a^	2.15^b^	0.43^bc^	1.65^a^	1.97^a^	0.26^a^
5 × 10^8^ CFU/kg	−	2.87^a^	1.41^a^	0.34^a^	2.00^ab^	2.04^a^	0.28^a^
10 × 10^8^ CFU/kg	−	3.15^a^	1.48^a^	0.49^c^	2.52^bc^	2.60^b^	0.34^b^
0	+	6.57^b^	3.58^1^	0.56^1^	3.01^1^	4.79^2^	0.41^c^
5 × 10^8^ CFU/kg	+	3.31^a^	2.80^c^	0.39^ab^	2.26^bc^	3.89^1^	0.37^b^
10 × 10^8^ CFU/kg	+	4.55^c^	2.29^bc^	0.46^bc^	2.34^bc^	3.07^c^	0.44^c^
SEM		0.25	0.16	0.02	0.09	0.20	0.01
Main effects							
*E. coli* challenge							
Negative		3.10^a^	1.68^a^	0.42^a^	2.05^a^	2.14^a^	0.29^a^
Positive		4.81^b^	2.95^b^	0.47^b^	2.57^b^	3.88^b^	0.41^b^
*L. acidophilus* supplementation							
0		4.90^a^	2.87^a^	0.51^a^	2.33	3.40^a^	0.340^a^
5 × 10^8^ CFU/kg		3.09^b^	2.20^b^	0.37^b^	2.13	2.84^b^	0.32^a^
10 × 10^8^ CFU/kg		3.85^c^	1.86^b^	0.47^a^	2.48	2.81^b^	0.39^b^
*P*-value^5^							
* E. coli* challenge		0.001	0.001	0.034	0.001	0.001	0.001
* L. acidophilus*		0.003	0.001	0.001	0.112	0.026	0.001
*E. coli* challenge × * L. acidophilus*		0.001	0.146	0.020	0.001	0.010	0.066

^a,b,c^Means within the same column with different superscripts differ significantly (*P* < 0.05).

1 Without *E. coli* challenge; +, with *E. coli* challenge.

2 CRP, diamine oxidase.

3 CRP, C-reactive protein.

4 LPS, lipopolysaccharide.

5 *P*-values represent the main effect of the *L.acidophilus*, the main effect of *E. coli* challenge, and the interaction between the dietary *L.acidophilus* and *E. coli* challenge.

The serum CRP, DAO, and LPS concentrations were considered as important symbols of intestinal permeability and barrier function. Main effects showed that dietary *L. acidophilus* supplementation reduced the serum concentration of CRP, DAO, and LPS in the serum at d 14 (*P* < 0.05). *E. coli* challenge significantly increased the serum concentration of CRP, DAO, and LPS (*P* < 0.05) compared with unchallenged birds and challenged birds fed with *L. acidophilus* over the whole infection period (14 d and 21 d).

### Inflammatory Cytokines mRNA Expression in the Jejunum and Spleen

Changes in the mRNA expression of inflammatory cytokines in the jejunum are shown in Table 7. At 21 d, the mRNA expression of iNOS, IL-8, and IL-1β were significantly (*P* < 0.05) upregulated by *E. coli* challenge, while *L. acidophilus* supplementation significantly (*P* < 0.05) downregulated the expression of IL-8. An interactive effect on the NF-kB mRNA expression (*P* < 0.05) in the jejunum was observed between *E. coli* challenge and *L. acidophilus* supplementation. In unchallenged groups, increasing *L. acidophilus* supplementation significantly (*P* < 0.05) downregulated the mRNA expression of NF-kB. However, the NF-kB mRNA expression was the lowest in *E. coli* challenged birds fed with lower dietary level of *L. acidophilus* (5 × 10^8^ CFU/kg).

**Table 7:** Effect of *L. acidophilus* on cytokine mRNA expressions in the jejunum of broilers infected with *E. coli* on d 21 (log2 relative).

*L. acidophilus*	*E. coli* challenge^1^	iNOS^2^	IL-6^3^	IL-8	IL-1β	NF-kB^4^
0	−	1.01^a^	0.96	1.17^a^	1.04^a^	0.95^ab^
5 × 10^8^ CFU/kg	−	1.02^a^	0.85	1.22^a^	1.17^ab^	1.25^b^
10 × 10^8^ CFU/kg	−	0.92^a^	0.76	1.12^a^	1.13^ab^	0.80^a^
0	+	1.78^b^	1.12	3.14^b^	1.88^b^	1.34^b^
5 × 10^8^ CFU/kg	+	1.43^ab^	0.71	1.16^a^	1.24^ab^	0.80^a^
10 × 10^8^ CFU/kg	+	1.16^a^	1.01	1.37^a^	1.91^b^	1.23^b^
SEM		0.09	0.10	0.20	0.12	0.07
Main effects						
*E. coli* challenge						
Negative		0.97^a^	0.87	1.12^a^	1.10^a^	1.02
Positive		1.46^b^	0.95	1.87^b^	1.68^b^	1.12
*L. acidophilus* supplementation						
0		1.39	0.78	2.06^a^	1.44	1.17
5 × 10^8^ CFU/kg		1.22	0.89	1.18^b^	1.20	1.03
10 × 10^8^ CFU/kg		1.04	0.86	1.23^b^	1.52	1.02
*P*-value^5^						
* E. coli* challenge		0.005	0.734	0.002	0.010	0.312
* L. acidophilus*		0.172	0.595	0.004	0.402	0.388
* E. coli* challenge × * L. acidophilus*		0.321	0.769	0.001	0.210	0.007

^a,b^Means within the same column with different superscripts differ significantly (*P* < 0.05).

1 Without *E. coli* challenge; +, with *E. coli* challenge.

2 iNOS, inducible nitric oxide synthase.

3 IL, interleukin.

4 NF-kB, nuclear factor kappa.

5 *P*-values represent the main effect of the *L. acidophilus*, the main effect of *E. coli* challenge, and the interaction between the dietary *L. acidophilus* and *E. coli* challenge.

*E. coli* challenge and *L. acidophilus* supplementation showed an interactive effect on the mRNA expression of iNOS (*P* < 0.05) in the spleen (Table 8). The expression of iNOS in the spleen was significantly (*P* < 0.05) upregulated by *E. coli* challenge, while *L. acidophilus* supplementation significantly (*P* < 0.05) downregulated the expression of iNOS of birds when challenged by *E. coli*. The relative mRNA expression levels of IL-8, IL-1β*,* and NF-kB in the spleen were not significantly affected by *E. coli*. challenge or by *L. acidophilus* supplementation (*P* > 0.05).

**Table 8:** Effect of *L. acidophilus* on cytokine mRNA expressions in the spleen of broilers infected with *E. coli* on d 21 (log2 relative).

*L. acidophilus*	*E. coli* challenge^1^	iNOS^2^	IL-6^3^	IL-8	IL-1β	NF-kB^4^
0	−	1.06^a^	0.98^ab^	1.04	1.12	1.10
5 × 10^8^ CFU/kg	−	1.17^ab^	1.14^b^	0.97	1.39	1.34
10 × 10^8^ CFU/kg	−	1.92^b^	0.68^a^	0.95	1.82	1.35
0	+	3.17^c^	1.02^ab^	0.90	0.68	1.35
5 × 10^8^ CFU/kg	+	1.22^ab^	0.81^ab^	1.06	1.22	1.37
10 × 10^8^ CFU/kg	+	1.21^ab^	0.87^ab^	1.17	0.85	1.32
SEM		0.19	0.06	0.09	0.17	0.08
Main effects						
*E. coli* challenge						
Negative (−)		1.362^a^	0.94	0.97	1.40	1.23
Positive (+)		1.864^b^	0.90	1.05	0.92	1.35
*L. acidophilus* supplementation						
0		2.08^a^	1.01	0.95	0.90	1.22
5 × 10^8^ CFU/kg		1.19^b^	0.97	1.01	1.31	1.39
10 × 10^8^ CFU/kg		1.56^ab^	0.78	1.07	1.34	1.37
*P*-value^5^						
* E. coli* challenge		0.037	0.726	0.721	0.178	0.521
* L. acidophilus*		0.014	0.197	0.898	0.442	0.665
* E. coli* challenge × * L. acidophilus*		0.001	0.169	0.802	0.616	0.642

^a,b^Means within the same column with different superscripts differ significantly (*P* < 0.05).

1 Without *E. coli* challenge; +, with *E. coli* challenge.

2 iNOS, inducible nitric oxide synthase.

3 IL, interleukin.

4 NF-kB, nuclear factor kappa.

5 *P*-values represent the main effect of the *L. acidophilus*, the main effect of *E. coli* challenge, and the interaction between the dietary *L.acidophilus* and *E. coli* challenge.

### Occludin, ZO-1, and Claudin mRNA Expression in the Jejunum and Ileum

No significant interaction effects were observed on occludin, ZO-1, and claudin mRNA expression in jejunum and ileum between *E. coli* challenge and *L. acidophilus* supplementation (Table 9). Compared with the control group and *E. coli* challenged broilers*, L. acidophilus* supplementation significantly upregulated the mRNA expression of occludin, ZO-1, and claudin in the jejunum, and occludin and ZO-1 in the ileum at 21 d (*P* < 0.05). *E. coli* challenge had no negative effect on the mRNA expression of occludin, ZO-1, and claudin mRNA expression in the jejunum (*P* > 0.05), but significantly downregulated the Occludin mRNA expression in the ileum (*P* < 0.05), and showed a reduced trend for ZO-1 mRNA expression (*P* < 0.10).

**Table 9:** Effect of *L. acidophilus* on mRNA expressions of Occludin, ZO-1, and Cladin-1 in the jejunum and ileum of broilers infected with *E. coli* on d 21 (log2 relative).

		Jejunum			Ileum		
*L. acidophilus*	*E. coli* challenge^1^	Occludin	ZO-1^2^	Cladin-1	Occludin	ZO-1	Cladin-1
0	−	0.94^a^	1.10^a^	1.05^a^	1.08^ab^	0.95^abc^	0.94^ab^
5 × 10^8^ CFU/kg	−	1.88^b^	1.39^ab^	1.72^abc^	1.37^c^	1.18^bc^	0.88^a^
10 × 10^8^ CFU/kg	−	1.81^b^	1.54^ab^	1.95^bc^	1.24^bc^	1.15^bc^	1.19^ab^
0	+	0.93^a^	1.04^a^	1.16^ab^	0.71^a^	0.76^a^	0.72^a^
5 × 10^8^ CFU/kg	+	1.88^b^	1.65^ab^	1.89^bc^	0.89^ab^	0.89^ab^	1.08^ab^
10 × 10^8^ CFU/kg	+	1.61^b^	1.92^b^	2.24^c^	1.11^bc^	1.24^c^	1.52^b^
SEM		0.11	0.12	0.14	0.06	0.05	0.09
Main effects							
*E. coli* challenge							
Negative		1.564	1.31	1.56	1.20a	1.11	1.02
Positive		1.478	1.54	1.76	0.91b	0.96	1.10
*L. acidophilus* supplementation							
0		0.97^a^	1.02^a^	1.08^a^	0.85^a^	0.88^a^	0.86^a^
5 × 10^8^ CFU/kg		1.88^b^	1.52^b^	1.81^b^	1.13^b^	1.03^ab^	0.98^a^
10 × 10^8^ CFU/kg		1.71^b^	1.74^b^	2.11^b^	1.18^b^	1.20^b^	1.35^b^
*P*-value^3^							
* E. coli* challenge		0.522	0.297	0.325	0.005	0.098	0.602
* L. acidophilus*		0.001	0.041	0.040	0.025	0.027	0.054
* E. coli* challenge × * L. acidophilus*		0.845	0.793	0.956	0.306	0.159	0.266

^a,b,c^Means within the same column with different superscripts differ significantly (*P* < 0.05).

1 Without *E. coli* challenge; +, with *E. coli* challenge.

2 ZO-1, zona occludens protein-1.

3 *P*-values represent the main effect of the *L. acidophilus*, the main effect of *E. coli* challenge, and the interaction between the dietary *L. acidophilus* and *E. coli* challenge.

## DISCUSSION

*Lactobacilli* can alleviate pathogenic inflammatory reactions and modulate the expression of key immune cytokines to improve host gut health and growth performance in poultry after pathogen exposure (Wang et al., 2017) and pig (Lee et al., 2012). The current study highlighted the significance of *L. acidophilus* supplementation in broiler production and intestinal health. Dietary *L. acidophilus* supplementation also increased the BW and ADG in broilers in a previous study (Wu et al., 2019). This indicated that *L. acidophilus* improved broiler growth*.* For *E. coli* challenged birds, the BW, ADG, and ADI were also improved with *L. acidophilus* supplementation compared with values in the control group. These results are consistent with other studies showing that the use of probiotic *Lactobacilli* positively improved the growth performance of pathogen challenged birds (Li et al., 2018; Redweik et al., 2019). The mechanism of probiotic action of *Lactobacilli* is multifactorial and not fully characterized. Possible mechanisms include secretion of antimicrobial substances, strengthening of the intestinal epithelial barrier function, competitive adherence to the mucosa, and modulation of the immune response (Broom and Kogut, 2018). *Lactobacilli* can prevent the growth of *E. coli* and *Salmonella* in vitro (Lin et al., 2008; Tejero et al., 2012), increase the *Lactobacillus* numbers in the ileum and cecum, and decrease the ileal *Escherichia* counts in vivo (Li et al., 2018). Improved intestinal flora may counteract the increased serum endotoxin content induced by intestinal pathogens. Fukata *et al*. (1988) reported that *L. acidophilus* decreased the toxins produced by intestinal pathogens, which explained the beneficial effect resulting from *L. acidophilus* addition. Growth improvement after *L. acidophilus* supplementation may also be due to its ability to produce endogenous enzymes or growth-promoting factors by directly fermenting nutrients in the gut. This could stimulate small intestine peristalsis, boost feed digestibility and availability, and promote gut health (Jin et al., 2000; Hung et al., 2012; Wu et al., 2019). Immunity stress can cause a mass of energy consumption to synthesize inflammatory factors, so it is possible that *L. acidophilus* reduced the immunity stress and preserved available energy for growth and maintenance.

Intestinal morphology, including villus height, crypt depth, and intestinal wall thickness, as well as the V/C ratio, are important parts of intestinal health and function (Wu et al., 2018). In this study, *L. acidophilus* supplementation of *L. acidophilus* in broiler diet increased the jejunum V/C ratio and ileum villus height, which is consistent with previous reports that *Lactobacilli* can improve the intestinal morphology development of broilers (Wang et al., 2012; Forte et al., 2017). The villus height and V:C ratio is meaningful indexes of the intestine absorptive ability and important parameters of healthy bird intestines (Jia et al., 2010; Li et al., 2018). In this study, the mortality rate of *E. coli* challenged birds was the highest among all groups examined. This indicated that *E. coli* challenge led to inflammatory responses and reduced the broilers intestinal health. Wu et al. (2018) reported that birds infected with necrotic enteritis (**NE**) pathogens had shorter villi height and decreased villus height to crypt depth V/C ratio in the jejunum. However, we found no remarkable differences on intestine villus height and V/C ratio in *E. coli* challenged birds. The specific strain of *E. coli* used in this study might not damage the host by disrupting the intestinal morphology.

Tight junctions are the most important components of the intestinal epithelial cell barrier function and intestinal permeability that protects the host from foreign pathogens and limits macromolecular transmission (Ballard et al., 1995). Tight junctions and intestinal permeability are formed by several types of proteins, including occludin, ZO-1, and claudin (Schneeberger, 2004). Wang et al. (2017) showed that *E. coli* infection greatly damaged the intestinal epithelial permeability and mucosal barrier function of broilers, resulting in gut pathogen translocation to liver and blood. We observed that *E. coli* challenge significantly increased the serum CRP and DAO concentration compared with unchallenged birds and challenged birds fed with *L. acidophilus* over the entire infection period. CRP is an acute phase protein and a component of the innate immune system. It is always regarded as a metabolic inflammatory marker (Wang et al., 2014; Bhattacharya et al., 2017). DAO is located mainly in the small intestinal mucosa. It is a marker for the assessment of gut barrier function, and enters the bloodstream when the gut barrier is impaired (Liu et al., 2020; Zhang et al., 2020). Increased serum CRP and DAO concentrations reflect changes in intestinal permeability, suggesting that *E. coli* challenge disrupts the intestinal barrier function. In addition, *E. coli* challenge downregulated the ileum barrier protein (occludin and ZO-1) mRNA expression and increased the jejunum wall thickness. Increased intestinal wall thickness is often accompanied with inflammatory bowel disease and affects the absorption and utilization of nutrients (Rudorf et al., 2010). Several studies demonstrated that *Lactobacilli* can inhibit intestinal pathogen growth and enhance poultry growth performance, by way of maintaining normal intestinal barrier function when faced with a pathogen challenge (Geier et al., 2010; Wang. et al., 2017). We found that the serum CRP and DAO levels of broiler chickens in the *L. acidophilus* groups were similar to the control group, regardless of *E. coli* challenge. *L. acidophilus* supplementation also significantly upregulated the mRNA expression of occludin, ZO-1, and claudin in the jejunum, and occludin and ZO-1 in the ileum, which corresponds to our results of lower serum LPS content. These results here indicated that dietary *L. acidophilus* supplementation reduced the intestinal damage caused by *E. coli* by improving the mRNA expression of intestinal barrier functional proteins. This is a significant finding that can be used to improve the intestinal health of broilers.

Host inflammatory responses are triggered when broilers are infected by pathogens from feed or the environment*.* LPS is the major component of the gram-negative bacteria cell wall and plays a key role in the inflammatory responses of hosts challenged by *E. coli* (Lim et al., 2016). If the pathogen-induced inflammatory response is severe, tissue damage can occur, accompanied by increased serum LPS content and reduced growth performance as was observed in the present study. In addition, due to *E. coli* infection induced cellular immunosuppression and infected birds had lower related total T (CD3+) cells counts than uninfected birds. Dietary *L. acidophilus* supplementation alleviated the decrease in CD3+ T cell counts caused by *E. coli* challenge, which is consistent with previous findings (Wang et al., 2015)*.* The T cell mediated immunity response plays a key role in the clearance of pathogens by influx into the intestine. The increase in circulating CD3+ T cell counts endowed the birds with a stronger immunity against *E. coli* or challenge from other pathogens. However, research on the effect of *L. acidophilus* on the cellular immunity of broilers is limited. The increase in intestinal T regulatory cells can be accelerated by *Lactobacillus* administration to animal diet (Liu et al., 2013; Meyer et al., 2020), which may explain how *L.* acidophilus increases the proportion of CD3+ cell in the blood of broilers as observed in the present study. The CD4+ (helper) and CD8+ (cytotoxic) T lymphocytes subsets are components of cell-mediated immunity and the stimulation of CD4+ and CD8+ T cells is necessary and essential in maintaining the cellular immune response in animals and humans when facing pathogen challenge (Ashraf and Shah, 2014). We found that increased CD4+ T cells counts in infected birds was a normal immune response and host self-protection process. Previous studies showed that *Lactobacilli* supplementation can increase the CD4+ and CD8+ T cells populations in broilers in the absence of pathogens challenge, indicating that *Lactobacillus* may benefit the host in maintaining the normal intestinal immune status and preventing disease invasion (Bai et al., 2013; Wang et al., 2015). However, these beneficial effects on CD4+ and CD8+ T cells of broilers were not observed in this study, which is contrary to previous reports. Similarly, Sefcova et al. (2020) also did not observe the benefit of CD4+ and CD8+ T cells counts when they fed *Lactobacillus fermentum* to broilers. This may have been caused by the difference between *Lactobacilli* strains. Wang et al. (2015) observed increased CD4+ T cells in broiler jejunum rather than in blood, suggesting that changes to lymphocyte populations by *Lactobacillus* are more likely to occur at local sites of colonization with minimal systemic effects.

The immune globulin is the first antibody isotype in the primary humoral immunity response. It is produced by mature B cells in response to antigen stimulation and co-stimulatory signals received from CD4+ T helper cells (Zhang et al., 2016). The immune globulin levels in serum are an indicator of long-term exposure to foreign antigen from feed or environment. We found that compared with unchallenged birds, *E. coli* challenge resulted in an increase in the serum IgA and IgM levels at the early stage of infection at d 14 (at 3 d postinfection), but decreased and were significantly lower than in unchallenged birds at 21 d (at 10 d postinfection). This is a classic phenomenon during the switch from acute to chronic inflammation. When broilers are in the early infection stage, *E. coli* acts as foreign antigen stimulate to B cells to regulate the immune system. Activated B cells then differentiate into plasmocytes and become plasma cells that secrete antibodies to neutralize viruses or become long-lived memory B cells (Chen et al., 2017). At the late infection stage, *E. coli* challenge induced cellular immunosuppression. Infected chickens showed lower circulating T cell counts and damaged cellular immune function, which was observed in the present study. We hypothesized that *E. coli* challenge led to B cell migration and impaired B-cell proliferation and viability, and then inhibited IgM and IgA secretion in the late infection stage. Previous studies showed that *Lactobacillus* administration increased levels of immune globulins such as IgA and IgM, suggesting that *L. acidophilus* presupplementation may have enhanced the defense ability of the broilers in the present study (Sefcova et al., 2020). Our data showed that *L. acidophilus* supplementation alone did not affect the serum IgA and IgM levels at 14 d, but produced large increases in serum IgA and IgM levels. IgA is widely distributed in the mucous secretions of the gastrointestinal and respiratory tracts and it helps prevent attachment of viruses and bacteria to epithelial surfaces and to neutralize toxins (Mccoy et al., 2008). These result may be due to a reduction in the number of pathogens in the intestinal tract of *E. coli* infected chickens fed *L. acidophilus* during the infection period, which was insufficient to stimulate the immune system to produce more specific immune globulin.

The total number of T-lymphocytes is tightly controlled by cytokines. Activated T-lymphocytes are able to act on inflammation through the secretion of IL-17, and stimulation of IL-8 and IL-6 expression (Kawasaki et al., 2009; Minmin et al., 2018). *Lactobacilli* were previously shown to regulate the transcript levels of inflammation related cytokines in previous studies (Kawasaki et al., 2009; Tan et al., 2015). Li et al. (2018) reported that *L. acidophilus* decreased the transcription of IL-8, IL-1β, and TNF-α in the spleen, and decreased the transcription of IL-8, IFN-γ, and IL-10 jejunum of broilers, irrespective of *Clostridium perfringens* challenge. Wang et al. (2015) showed that a *L. plantarum* strain increased the jejunal transcription of IFN-γ, IL-12, and IL-4 at 14 d post-treatment in the absence of pathogens. These studies demonstrated that *Lactobacilli* can increase host immunity in the presence or absence of pathogens. However, little is known about the effects of *L. acidophilus* on the cytokine expression under an *E. coli* challenge model. In the present study, *L. acidophilus* supplementation decreased the transcription of IL-8, iNOS and IL-1β in the jejunum, and decreased the transcription of iNOS in the spleen of *E. coli* challenged broilers. We also observed differences in cytokine expression of *E. coli* challenged birds in the jejunum and spleen. This may be because the inflammatory responses appeared more intense in the jejunum than in the spleen, which might lead to intestinal damage, higher nutrient consumption, and poor growth performance.

IL-6 is involved in several immune responses. The main function of IL-6 is to activate B cells to proliferate and secrete antibodies, stimulate T cell proliferation, and activate cytotoxic T lymphocytes (Zhou et al., 2019). Tan et al. (2015) found that IBDV inoculation reduced broiler serum IL-6 concentration. The result may have involved either innate or adaptive immunity and was illustrated by immunosuppression. However, no significant changes were found on IL-6 gene expression, both in jejunum and spleen, in the present study. This may be due to the differences of the infection model. *Lactobacilli* exhibit different regulatory functions when hosts are under different conditions. IL-8 acts as a chemotactic for neutrophils and monocytes in inflammation. The upregulation of IL-8 mRNA expression is a protective response of chickens for the clearance of invading host pathogens. In the present study, *E. coli* challenge upregulated the mRNA expression of jejunum IL-8 and this was similar to previous results (Sreedhar et al., 2008). These findings indicate that dietary *L. acidophilus* supplementation enhanced the immunity of chickens against *E. coli* challenge. Macrophages can produce proinflammatory cytokines IL-1*β* during the inflammatory responses, and mediate the inflammation at the early infection period. Local inflammatory response recruits phagocytic and non-phagocytic lymphoid cells to remove foreign pathogens. The upregulation expression of IL-1β during *E. coli* infection is critical for birds through the clearing of foreign pathogens. The iNOS has a synergistic effect with some inflammatory mediators, which may cause cellular swelling and apoptosis (Zhang et al., 2017). NO is an important inflammatory product produced by iNOS, and it is mainly involved in promoting inflammatory responses(Xu et al., 2017). NO typically acts by inhibiting the inflammatory response. Under pathological conditions, the iNOS synthesizes a large amount of NO, which has a cytotoxic effect and aggravates the inflammatory response. In this study, dietary *L. acidophilus* (10 × 10^8^ CFU/kg) supplementation increased the mRNA expression of jejunum. Therefore, *L. acidophilus* may enhance the host defense function by increasing NO production in the intestinal epithelium of broilers.

## CONCLUSIONS

The protective effects of dietary *L. acidophilus* supplementation on *E. coli* challenged birds resulted from an enhancement in cellular and humoral immunity and improvement in intestinal barrier function. These effects resulted in improved growth performance and reduced mortality of birds. *L. acidophilus* can be used as an intervention strategy to reduce *E. coli* infection in broilers. However, the mechanism by which dietary *L. acidophilus* supplementation exerts protective effects against intestinal microflora and intestinal diseases requires further study.

## DISCLOSURES

The authors declare no conflicts of interest.
